# Effects of *Epichloë gansuensis* Endophyte on the Root and Rhizosphere Soil Bacteria of *Achnatherum inebrians* Under Different Moisture Conditions

**DOI:** 10.3389/fmicb.2020.00747

**Published:** 2020-04-17

**Authors:** Yawen Ju, Rui Zhong, Michael J. Christensen, Xingxu Zhang

**Affiliations:** ^1^State Key Laboratory of Grassland Agro-ecosystems, Center for Grassland Microbiome, Key Laboratory of Grassland Livestock Industry Innovation, Ministry of Agriculture and Rural Affairs, Engineering Research Center of Grassland Industry, Ministry of Education, College of Pastoral Agriculture Science and Technology, Lanzhou University, Lanzhou, China; ^2^Grasslands Research Centre, AgResearch, Palmerston North, New Zealand

**Keywords:** *Epichloë gansuensis*, soil moisture, Achnatherum inebrians, bacterial diversity, plant-microbe interaction

## Abstract

This study was conducted to explore effects of the systemic fungal endophyte *Epichloë gansuensis* on root and rhizosphere soil bacterial diversity of *Achnatherum inebrians* host plants growing under different moisture conditions. Soil properties of different treatments were compared using standard techniques. A total of 4371379 16S rRNA gene sequences were obtained and assigned to 5025 operational taxonomic units (OTUs). These OTUs in roots and rhizosphere soil were divided into 13 and 17 phyla, respectively, and the Actinobacteria and Proteobacteria were the most abundant phyla both in roots and rhizosphere soil. Shannon diversity and Chao1 richness index of bacteria in rhizosphere soil was significantly higher than in roots. *E. gansuensis* decreased the Shannon diversity of the root-associated bacterial community, and increased Shannon diversity and Chao1 richness index of the rhizosphere soil bacterial community of *A. inebrians*. Meanwhile, Chao1 richness of the rhizosphere soil bacterial community of *A. inebrians* significantly increased with the increase of the soil moisture level. Structural equation modeling also emphasized that *E. gansuensis* decreased the diversity of the root-associated bacterial community and increased the diversity of the rhizosphere soil bacterial community through decreasing soil available *N*. Additionally, soil moisture increased the diversity of the rhizosphere soil bacterial community through increased soil pH, C/N, and NN, and decreased soil AP. The *E. gansuensis* endophyte and soil moisture effects on root and rhizosphere soil bacterial diversity were likely to be from responses to modifications of the rhizosphere soil properties.

## Introduction

Microbial associations are widely distributed in terrestrial ecosystems, and plant tissues are associated with a wide range of microbes, including fungi (Johnson et al., 2013), and bacteria (Bell-Dereske et al., 2017). Endophytic fungi belonging to the genus *Epichloë* have been found in many cool-season grasses (Schardl et al., 2004; Leuchtmann et al., 2014). The associations between host grasses and the genus *Epichloë* are generally considered to be mutualistic and the transmission of many species is completely vertical (Schardl et al., 2004; Christensen et al., 2008). Previous studies on these symbiotic relationships have focused on the genera *Lolium* and *Festuca* because they enhance the adaptability and productivity of host plants under abiotic and biotic stresses (Johnson et al., 2013; Soto-Barajas et al., 2016).

Another grass species that is host to an *Epichloë* endophyte and which has become the focus for intense research is *Achnatherum inebrians.* This is a widespread perennial bunchgrass in the Qinghai-Tibet Plateau, including Tibet, Qinghai, Xinjiang, and Gansu provinces (Nan and Li, 2000; Li et al., 2004). Nearly 100% of *A. inebrians* plants in these regions are host to an *Epichloë* endophyte (Nan and Li, 2000). The endophyte was originally classified as being a *Neotyphodium* species as the sole method of transmission is through the seed of host plants; stromata in which the sexual stage of some *Epichloë* species is formed have never been observed, almost certainly eliminating the possibility of horizontal transmission. The endophyte of *A. inebrians* was originally given the name of *Neotyphodium gansuense* (Li et al., 2004) but was later classified as *E. gansuensis* (Leuchtmann et al., 2014). A subsequent study revealed that *A. inebrians* plants originating from seed obtained from seven of eight populations from the grasslands of Xingjiang, Gansu and Inner Mongolia provinces of China were host to a distinctive *Epichoë* endophyte that was given the name *E. inebrians* (Chen et al., 2015). *A. inebrians* plants have long been associated with a narcotic-type effect on grazing livestock, giving rise to the species name *inebrians* and the common name of drunken horse grass (Zhang et al., 2011, 2014a; Liang et al., 2017). In 1996, high levels of ergonovine and lysergic acid amide were identified in leaves of *A. inebrians* plants infected with a systemic endophyte, then referred to as an *Acremonium* sp of the *albo-lanosa* section Morgan-Jones and Gams (Miles et al., 1996). It is these endophyte-produced alkaloids that are present in *A. inebrians* plants host to *E. inebrians* (Chen et al., 2015). *A. inebrians* plants host to *E. gansuensis* were found in that study to contain the indole-diterpene alkaloid paxilline (Chen et al., 2015). The presence of an *Epichloë* endophyte in *A. inebrians* plants provides enhanced tolerance to abiotic stresses including drought stress (Xia et al., 2018), salt stress (Wang et al., 2018), heavy metals (Zhang et al., 2010), and low temperature (Chen et al., 2016), and as well as resistance to fungal pathogens (Xia et al., 2016) and insect pests (Zhang et al., 2012). The deterrence of grazing and the enhanced tolerance to abiotic and biotic stresses conferred by the presence of an *Epichloë* endophyte have led to the greatly increased distribution of *A. inebrians* throughout the grasslands of northwest China that have been degraded by overgrazing (Zhao et al., 2005; Yao et al., 2015).

Effects of *Epichloë* endophytes, hyphae of which are absent in roots, have been reported on belowground organisms, especially microorganisms, and under different ambient conditions (Rojas et al., 2016; Bell-Dereske et al., 2017; Zhong et al., 2018). Included in the microorganisms affected are arbuscular mycorrhizal fungi (AMF; Rojas et al., 2016) and phosphorus-solubilizing fungi (Arrieta et al., 2015). Additionally, previous studies also found that the presence of an *Epichloë* endophyte decreased the abundance of gram-positive bacteria in soil of tall fescue (*Festuca arundinacea*; Buyer et al., 2011) and the root-associated bacterial diversity of American beachgrass (*Ammophila breviligulata*; Bell-Dereske et al., 2017), while increased the rhizosphere soil bacterial diversity associated with tall fescue (Roberts and Ferraro, 2015). The presence of an *Epichloë* endophyte in annual ryegrass (*Lolium multiflorum*) changed the composition of the soil bacterial community (Casas et al., 2011).

The composition and diversity of bacterial communities in soil and roots are considered as indicators reflecting plant biomass, mineral resources acquisition and biological processes, which are inevitably affected by host plant and environmental factors, including pH (Shen et al., 2013), nutrient availability (Meyer et al., 2013), soil moisture (Zhang et al., 2014b), and fertility (Yao et al., 2018). These factors alter rhizosphere soil microbial communities by changing soil physical and chemical properties, nutrient cycling and phytohormones production (Zhang et al., 2014b; Francioli et al., 2017; Zhalnina et al., 2018). Previous research also indicated that plant genotype and vegetation growth stage transforms the plant bacterial diversity, and which can promote release of secondary metabolites from roots, influencing microbial diversity and community composition in rhizosphere soil (Guo et al., 2015; Vandegrift et al., 2015; Rojas et al., 2016; Soto-Barajas et al., 2016).

A previous study had found that the presence of *Epichloë gansuensis* increased the spore diversity of AMF in the *A. inebrians* plants rhizosphere soil under different growth conditions (Zhong et al., 2017), and decreased the root-associated fungal diversity under cultivation (Zhong et al., 2018). However, how *E. gansuensis* affects the bacterial diversity of rhizosphere soil and roots of *A. inebrians* is poorly understood. Our previous study had indicated that the presence of *E. gansuensis* could markedly improve water-use efficiency of *A. inebrians* plants under limited soil water content in greenhouse conditions (Xia et al., 2018). Furthermore, *E. gansuensis* also promoted the growth and development of *A. inebrians* roots under low soil moisture in the field (Xia, 2018). However, the effects of soil moisture on bacterial diversity in rhizosphere soil and roots of *A. inebrians* have not been reported. To address these questions, the objective of this present study was to investigate effects of *E. gansuensis* on bacterial diversity of rhizosphere soil and roots of *A. inebrians* plants under different moisture conditions. It was hypothesized that (1) *E. gansuensis* and soil moisture levels could influence the bacterial diversity in roots of *A. inebrians* plants and rhizosphere soil, (2) Changes in bacterial diversity in roots of *A. inebrians* plants and rhizosphere soil associated with the presence of *E. gansuensis* and changes in soil moisture may be related to the soil physical and chemical properties.

## Materials and Methods

### Site Description and Experimental Design

This study was conducted in field plots at the Yuzhong campus (104°39′E, 35°89′N, and attitude 1653 m) of the College of Pastoral Agriculture Science and Technology of Lanzhou University. The *A. inebrians* plants used in this study originated locally, from the location where the endophyte species present in this species of grass was assigned the name *N. gansuense* (Li et al., 2004). The endophyte infection status of 20 tillers from individual *A. inebrians* plants originating from that location was determined by aniline blue staining of leaf sheathes and observing under a microscope, and then in 2011, seeds were collected from the tillers of one *A. inebrians* plants with 100% endophyte-tiller infection (Li et al., 2016). Before planting in 2012, the collected seeds were divided into two parts, with one part treated with thiophanate methyl fungicide to eradicate *E. gansuensis*, while the other part was untreated. EF and EI *A. inebrians* seeds were planted separately at Yuzhong campus as described by Zhong et al. (2019). In 2013, seeds of *E. gansuensis*-free (EF) and *E. gansuensis*-infected (EI) *A. inebrians* plants were collected, the endophyte-infection status was confirmed in the laboratory, and then the seeds were stored at 4°C in a refrigerator. In 2014, EF and EI *A. inebrians* plants were established in the field, using seeds that had been collected from plants grown from seeds obtained from single EF and EI plants originating from the same population to lower variability of the plants used in experiments, as described by Xia et al. (2018). Before planting, 50 seeds were randomly selected from EI and EF seed stocks to determine their endophyte-infection status and confirm that seed stocks used in the study were 100% and 0% infected, respectively. There were nine plots (each plot: 4.0 m × 4.8 m), and each was divided equally into two parts by a cement wall. EF and EI *A. inebrians* plants were planted individually in 4 lines and 8 rows.

From May to October of 2014–2016, three water treatments were maintained on split plots including three replicate plots. The first treatment was maintained at normal water content (N) and only received natural precipitation, the annual precipitation from 2014 to 2016 was 321 mm, 282 mm, and 256 mm, respectively. The second treatment was drought stress (D), in which the plots were manually covered. The third treatment was the sufficient irrigation condition (W), which received water every 3 days by overhead automatic irrigation, and to maintain 45–60% of the relative saturated soil moisture.

### Sample Description

Root and rhizosphere soil samples were collected at the end of water treatments in October 2016. For each sub-plot, roots and rhizosphere soil were obtained from 20 cm cores from five separate *A. inebrians* plants and following screening, the roots and rhizosphere soil were mixed to form mixed roots or rhizosphere soil samples. The 18 root and 18 rhizosphere soil samples were cooled and brought back to the laboratory. The root samples were gently rinsed several times with tap water then washed with sterile water, followed by drying on sterilized filter paper. Samples of these roots were stored at −80°C before DNA extraction. Before soil chemical analysis, soil samples were screened using a 2.0 mm sieve and stored at 4°C, while others were stored at −80°C.

### Soil and Biological Properties

Soil pH was analyzed at a ratio of 1:2.5 in soil/water mixtures. A Shimadzu total organic carbon (TOC)-VCPH analyzer was used to analyze TOC and total carbon (TC). According to the method of Nelson and Sommers (1982), 0.25 mm – sieved soil was used to measure the soil organic matter. Ammonium acetate and Flame Photometry were used to extract and analyze available potassium (AK; Helmke and Sparks, 1996). A molybdenum blue method was used to calculate the plant available phosphorus (AP; Robertson et al., 1999). A continuous flow analyzer (FIAstar 5000 Analyzer) was used to measure total nitrogen (TN), nitrate-N (NN), ammonium-N (AN), total P (TP), and available N in the soil (Zhao et al., 2014).

### DNA Extraction, Amplification, and Sequencing

Total DNA was extracted from approximately 0.1 *g* and 0.5 *g* of root and rhizosphere soil samples, respectively, by using a plant DNA kit (Tiangen, Beijing) and a Soil DNA Kit (OMEGA, Shanghai) according to the manufacturer’s instructions. Bacteria 16S rRNA genes were amplified by the primer pair of Eub518 (5′-ATT ACC GCG GCT GCT GG-3′) and Eub338 (5′-ACT CCT ACG GGA GGC AGC AG-3′). Two different thermostable DNA polymerases were used in the 16S rDNA PCR amplifications for each sample in order to reduce PCR bias: (I) Phusion High-Fidelity DNA Polymerase (Thermo Scientific, Sweden): 98°C for 2 min followed by 30 cycles of 98°C for 30 s (denaturation), 56°C for 20 s (annealing), 72°C for 20 s (polymerization), and a final extension at 72°C for 10 min, and confirmed the size of amplified product was appropriate. Using 1% agarose gels to mixed and visualized each DNA samples after electrophoresis. Then PCR products were purified with a kit (MO BIO Laboratories, Inc., Carlsbad, CA, United States), and submitted to Majorbio Pharm Technology (Shanghai, China) on the Illumina pyrosequencing^1^ for sequences.

### Bioinformatic Analyses

Pyrosequencing reads were assembled and filtered, and reads with ambiguous nucleotides, a quality score of less than 20, lacking complete barcode and primer were deleted and excluded from further analysis, and then the primer region was removed. The remaining sequences were assigned to operational taxonomic units (OTUs) using QIIME, requiring at least 97% threshold over at least 90% of the sequence length.^2^ These sequences were performed on the Silva database^3^ to identity these OTUs which were obtained from Illumina pyrosequencing. After removing the non-bacteria OTUs, the abundance information of the OTUs was normalized using the sequence number standard, which corresponded to the sample with the minimum sequence, and the rarefaction curves were generated based on these OTUs. Subsequent analysis of alpha and beta diversity is based on this output of standardized data.

### Alpha and Beta Diversity Analysis

Community richness was determined with the Chao1 index (Chao and Bunge, 2002) and community diversity was determined by the Shannon index (*H*′; Shannon, 1949), respectively, which was calculated using the formula.

$$\mathrm{Chao1}=S_{o\,b\,s}+\frac{F_{1}\,\left(F_{1}-1\right)}{2\,\left(F_{2}+1\right)}$$

Where the *S*_*obs*_ which represented the number of observed OTUs, and *F*_1_ and *F*_2_ are the number of singletons and doubletons, respectively.

$$H^{\prime }=-\sum_{n=1}^{s}\left(P_{i}\,l\,o\,g_{2}\,P_{i}\right)$$

Where *s* is the number of OTUs and *P*_*i*_ is the proportion of the bacteria community represented by the OTUs.

For diversity analysis, dissimilarity of *A. inebrians* plant root and rhizosphere soil bacterial communities were calculated using principal-coordinate analysis (PCoA) by pairwise analysis, which was performed using *R* software (version 2.14.0) by pairwise Bray–Curtis dissimilarity. Based on Bray–Curtis distances, analysis of similarity (ANOSIM) and permutational multivariate two-way analysis of variance (PERMANOVA) were performed to calculate the statistically significant differences of root and rhizosphere soil bacterial communities under different treatments. Redundancy analysis (RDA) among *A. inebrians* plant root and rhizosphere soil bacterial community abundance at the phylum level and rhizosphere soil properties, were conducted by CANOCO for Windows 4.5.

### Statistical Analyses

These differences of rhizosphere soil properties, and root and rhizosphere soil bacterial community diversity under different endophyte and soil moisture levels were tested using two-way analysis of variance (Two-way ANOVA) by SPSS 22.0 (SPSS Inc., Chicago, IL, United States). Significant differences among different soil moisture levels were tested by one-way analysis of variance (One-way ANOVA). Fishers least significant differences (LSD) test was used to determine whether differences between means were statistically significant. In all tests, *P*-value < 0.05 was considered statistically significant.

### Structural Equation Modeling

Structural equation modeling (SEM) was used to identify potential causal relationships between explanatory variables and bacterial diversity. According to the results of linear regression, we calculated the degree of intimacy of direct and indirect relationships between variables, and checked the binary relationship between variables to ensure the appropriateness of the linear model. Based on the potential relationship between known factors and driving factors of bacterial diversity, SEM models were constructed. χ^2^ test was used to evaluate the fitting of each model. In addition, AMOS 24.0 (Amos Development Co., Greene, MD, United States) was used for performing SEM analysis and others statistical analyses were calculated by SPSS 22.0 (SPSS, Inc., Chicago, IL, United States).

## Results

### Root and Rhizosphere Soil Bacterial Community Composition

**FIGURE 1:**
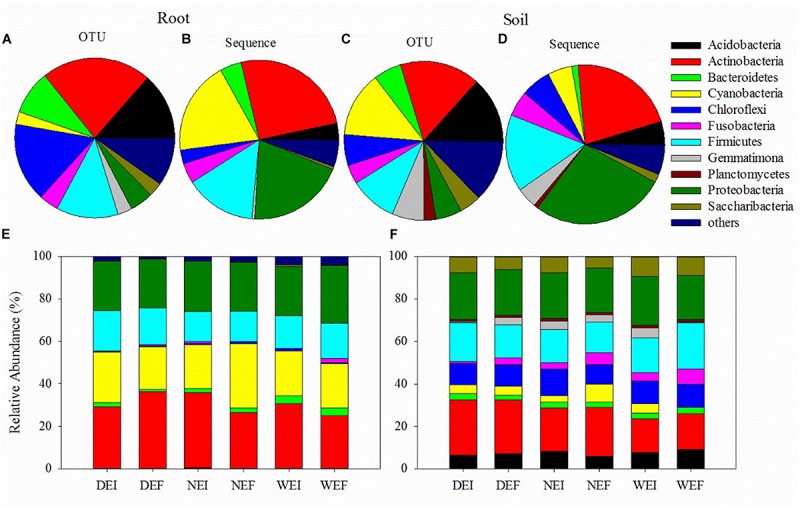
The bacterial community structures (at the phyla level) of samples from roots **(A,B)** and rhizosphere soil **(C,D)** using plant and soil DNA kits, as well as the composition of different phyla of bacteria from the (**E**) roots and (**F**) rhizosphere soil of *A. inebrians* on account of the classification of partial 16S rRNA sequences (*n* = 3, D: drought, N: normal, W: well-watered, EI: endophyte-infected, and EF: endophyte-free).

Using the pair of primers, a total of 1876756 and 2494623 sequences were obtained from rhizosphere soil and root samples, respectively (Figure 1 and Supplementary Table S1). There were 4994 OTUs detected in rhizosphere soil and 2627 OTUs detected in roots, and 2596 OTUs were present in both the roots and the rhizosphere soil. These OTUs in roots and rhizosphere soil were divided into 13 phyla and 17 phyla, respectively (Figure 1 and Supplementary Table S1). The bacterial communities in the roots of the three treatments harbored relatively fewer phyla compared to those in rhizosphere soil bacterial communities (Figure 1 and Supplementary Table S1). In addition, the overall patterns of relative abundance of the main groups at the phylum level in roots and rhizosphere soil is different among different endophyte plus soil moisture treatments (Figure 1 and Supplementary Table S1).

Actinobacteria (583 OTUs, 25.21% sequences) was the most abundant phylum in roots, while Proteobacteria (268 OTUs, 27.54% sequences) was the dominant phylum in rhizosphere soil bacterial communities under different treatments (Figures 1E,F and Supplementary Table S1). The following four most abundant phyla in roots were Proteobacteria (124 OTUs, 19.84% sequences), Cyanobacteria (67 OTUs, 18.86% sequences), Firmicutes (329 OTUs, 14.44% sequences), and Bacteroidetes (235 OTUs, 4.33% sequences; Figures 1B,D and Supplementary Table S1). In contrast, in rhizosphere soil the four next most abundant phyla were Actinobacteria (814 OTUs, 21.68% sequences), Firmicutes (483 OTUs, 15.68% sequences), Chloroflexi (314 OTUs, 6.07% sequences), and Fusobacteria (192 OTUs, 5.12% sequences; Figures 1B,D and Supplementary Table S1).

**FIGURE 2:**
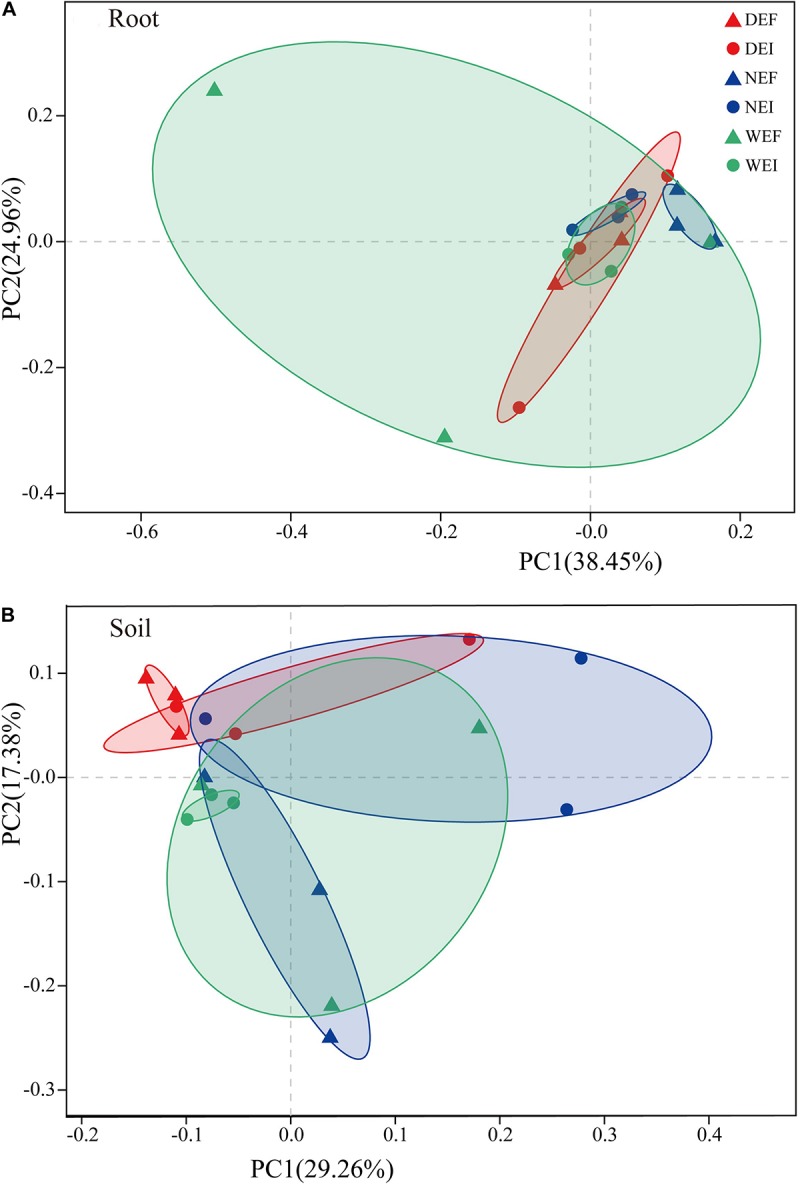
The structure of plant-related bacterial communities. Principal Coordinates analysis (PCoA) of pairwise Bray–Curtis dissimilarity between **(A)** root and **(B)** rhizosphere soil comparison within each of the three datasets tested with randomly sampled microbial community (*n* = 3, D: drought, N: normal, W: well-watered, EI: endophyte-infected, and EF: endophyte-free).

Rarefaction curves were generated for all root and rhizosphere soil treatments by using a 97% identity cutoff, which was used to depict the bacterial richness among different root (Supplementary Figure S1A) and rhizosphere soil samples (Supplementary Figure S1B). As the results show, the species of the bacterial community in roots (Supplementary Figure S1A) were less diverse than in rhizosphere soil (Supplementary Figure S1B). Principal coordinates analysis indicted that the root-associated and rhizosphere soil bacterial community composition between EI and EF *A. inebrians* differed among the *D*, *N*, and *W* treatments (Figures 2A,B and Table 1). *E. gansuensis*, soil moisture and their interactions had no significant (*P* > 0.05) effects on the diversity of the bacterial community in *A. inebrians* rhizosphere soil (Table 1), while, *E. gansuensis*, soil moisture and their interactions had significant (*P* < 0.05) effects on root-associated diversity of the bacterial community of *A. inebrians* (Table 1).

**TABLE 1:** The statistical test of similarity (ANOSIM) and permutational multivariate two-way analysis of variance (PERMANOVA) to analyze differences of *Achnatherum inebrians* root and rhizosphere soil bacterial community composition calculated by Illumina sequencing.

**Type**	**Treatment**	**df**	**PERMANOVA**		**ANOSIM**	
			**Bray–Curtis**		**Bray–Curtis**	
			***F***	***P***	***R***	***P***
Soil	E	1	0.7429	0.6102	−0.0823	0.4929
	W	2	0.4314	0.7612	−0.0247	0.5542
	W*E	2	0.7324	0.6264		
Root	E	1	5.0327	**0.0058**	0.5185	**0.0055**
	W	2	5.7199	**0.0045**	0.3251	**0.0115**
	W*E	2	5.5094	**0.0052**		

*Bold values indicate significant differences.*

### Root and Rhizosphere Soil Bacterial Community Diversity

**FIGURE 3:**
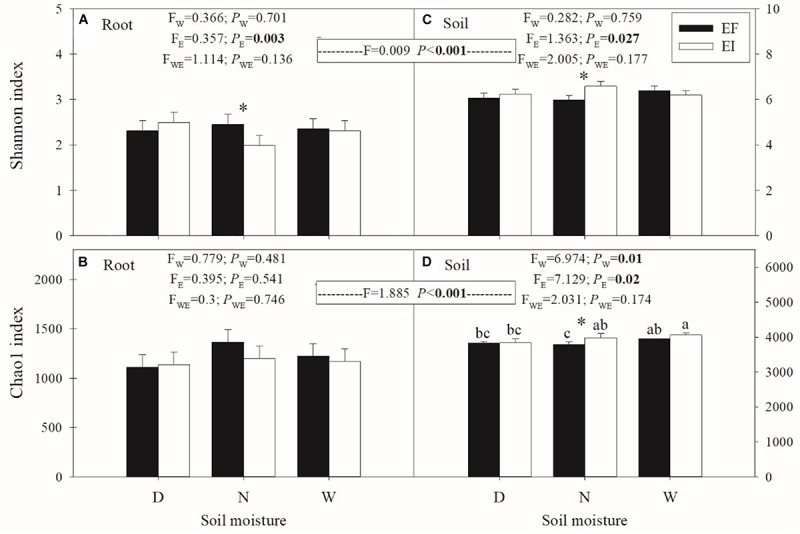
Bacterial community diversity in roots **(A,C)** and soil **(B,D)** under different water and endophyte treatments (*n* = 3, D: drought, N: normal, W: well-watered, EI: endophyte-infected, and EF: endophyte-free). Values are mean ± standard error (SE), with bars indicating SE. The asterisk (*) means significant difference at *P* < 0.05 (independent *t*-test) between EI and EF plants at corresponding water content. The A, B means significant difference at *P* < 0.05 among corresponding water content and endophyte status.

The results indicated that the Shannon diversity and Chao1 richness index of bacterial diversity in rhizosphere soil (*F* = 1.885; *P* < 0.001) was significantly higher than in roots (*F* = 0.009; *P* < 0.001; Figure 3). Furthermore, the presence of *E. gansuensis* significantly (*P* = 0.003) decreased the Shannon diversity of the bacterial community in roots of *A. inebrians* (Figure 3A). The interactions between *E. gansuensis* and soil moisture had no significant effects on the Shannon diversity and Chao1 richness of bacterial diversity in roots of *A. inebrians* (Figures 3A,B).

The presence of *E. gansuensis* significantly increased the Shannon diversity (*P* = 0.027) and Chao1 richness (*P* = 0.02) of the rhizosphere soil bacterial community of *A. inebrians* (Figures 3C,D). Meanwhile, compared to the normal soil moisture, drought markedly decreased the Chao1 richness of the rhizosphere soil bacteria community of *A. inebrians*, and the well-watered treatment significantly increased the Chao1 richness of the rhizosphere soil bacteria communities (Figure 3D). The interactions between *E. gansuensis* and soil moisture had no significant effects on the Shannon diversity and Chao1 richness of rhizosphere soil bacterial community of *A. inebrians* (Figures 3C,D).

### Relationship Between Bacteria and Soil Properties

**FIGURE 4:**
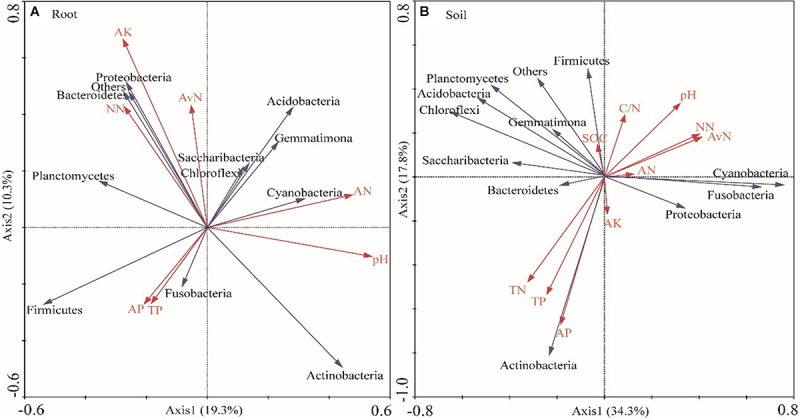
Redundancy analysis (RDA) of relative abundance of root **(A)** and rhizosphere soil **(B)** bacterial communities and soil properties under different water and endophyte treatments (*n* = 3, D: drought, N: normal, W: well-watered, EI: endophyte-infected, and EF: endophyte-free). Soil factors indicated include AN (Ammonium Nitrogen), NN (Nitrate Nitrogen), TN (Total Nitrogen), TP (Total Phosphorus), AP (Available P), AK (Available potassium), pH, SOC (Soil Organic Carbon), and Available N.

The soil moisture had significant (*P* < 0.01) effects on soil properties, especially AN, NN, TN, AP, and available N (Table 2). *E. gansuensis* had significant (*P* < 0.01) effects on soil properties, especially NN, AP, and available N (Table 2). The interactions between *E. gansuensis* and soil moisture had significant (*P* < 0.01) effects on *A. inebrians* rhizosphere soil AN and NN (Table 2). Spearman correlations results revealed that the Chao1 index of the *A. inebrians* rhizosphere soil bacterial community was positively and significantly (*P* < 0.05) associated with rhizosphere soil NN, available N, and N/P (Table 3), and negatively correlated with rhizosphere soil AP (Table 3). The Shannon index of the *A. inebrians* rhizosphere soil bacterial community had no significant (*P* > 0.05) correlation with rhizosphere soil properties (Table 3). Additionally, according to the RDA between the rhizosphere soil bacterial community and soil properties, the first and second axis of RDA explained 34.3% and 17.8% of the variance, respectively, as the length of each arrow represents the contribution of parameters to structural variation (Figure 4B). In addition, Proteobacteria was positively associated with rhizosphere soil AK, pH, AN, NN, and available N, while negatively associated with rhizosphere soil SOC and TN (Figure 4B). Meanwhile, Actinobacteria was positively associated with rhizosphere soil AK, AP, TP, TN, and negatively associated with rhizosphere soil pH, AN, NN, available N, SOC, and C/N (Figure 4B).

**TABLE 2:** The chemical properties of root-associated and rhizosphere soil bacterial community composition of *A. inebrians* under different soil moisture and endophyte treatments at phylum levels (*n* = 3, D: drought, N: normal, W: well-watered, EI: endophyte-infected, and EF: endophyte-free).

**Treatment**	**AN (mg/Kg)**	**NN (mg/Kg)**	**TN (%)**	**TP (%)**	**AP (mg/Kg)**	**AK (mg/Kg)**	**pH**	**SOC (%)**	**Available N (mg/Kg)**
DEI	3.384 ± 0.145	4.987 ± 0.188	0.060 ± 0.001	0.074 ± 0.002	7.734 ± 0.179	20.463 ± 1.210	7.993 ± 0.062	0.621 ± 0.023	8.373 ± 0.315
DEF	4.398 ± 0.064	6.144 ± 0.156	0.059 ± 0.000	0.076 ± 0.000	6.553 ± 0.084	16.342 ± 1.169	8.061 ± 0.086	0.601 ± 0.037	10.542 ± 0.216
NEI	5.250 ± 0.128	4.291 ± 0.350	0.058 ± 0.001	0.075 ± 0.003	6.512 ± 0.083	16.016 ± 1.779	8.014 ± 0.068	0.639 ± 0.006	9.539 ± 0.410
NEF	4.726 ± 0.097	7.662 ± 0.083	0.057 ± 0.001	0.074 ± 0.001	5.786 ± 0.315	17.292 ± 2.089	8.022 ± 0.064	0.632 ± 0.034	12.391 ± 0.017
WEI	4.061 ± 0.110	6.18 ± 0.308	0.061 ± 0.001	0.077 ± 0.001	5.381 ± 0.159	20.161 ± 0.565	8.041 ± 0.075	0.614 ± 0.064	10.232 ± 0.336
WEF	4.393 ± 0.153	7.889 ± 0.184	0.061 ± 0.000	0.074 ± 0.003	5.118 ± 0.152	17.609 ± 1.461	8.070 ± 0.052	0.650 ± 0.022	12.264 ± 0.312
E	0.067	**0.000**	0.160	0.125	**0.000**	0.142	0.543	0.885	**0.000**
W	**0.000**	**0.000**	**0.005**	0.165	**0.000**	0.285	0.861	0.859	**0.000**
E*W	**0.000**	**0.001**	0.143	0.430	0.061	0.183	0.848	0.650	0.337

*Values are mean ± standard error (*n* = 3). Soil factors indicated include AN, Ammonium Nitrogen; NN, Nitrate Nitrogen; TN, Total Nitrogen; TP, Total Phosphorus; AP, Available P; AK, Available potassium; pH, SOC, Soil Organic Carbon; and Available N. Bold values indicate significant differences.*

**TABLE 3:** Correlations between chemical properties in root or rhizosphere soil of *A. inebrians* under different soil moisture and endophyte treatments with alpha diversity was analyzed by Pearson’s correlation coefficient.

**Soil properties**	**Root**		**Soil**	
	**Shannon**	**Chao1**	**Shannon**	**Chao1**
AN	0.112	0.295	0.01	–0.094
NN	–0.23	–0.14	0.387	0.742**
TN	–0.042	0.116	–0.162	–0.201
TP	–0.064	0.095	–0.038	−0.561*
AP	0.093	–0.133	–0.292	−0.680**
AK	–0.074	–0.445	0.19	–0.044
pH	0.275	0.35	–0.085	0.164
SOC	0.172	0.366	0.13	0.304
C/N	0.185	0.312	0.171	0.347
Available N	–0.162	–0.007	0.352	0.630**
N/P	–0.167	0.065	0.339	0.758**

The asterisk indicates significant differences at the 0.01 and 0.05 level (**P < 0.01; *P < 0.05). Soil factors indicated include AN, Ammonium Nitrogen; NN, Nitrate Nitrogen; TN, Total Nitrogen; TP, Total P; AP, Available P; AK, Available potassium; pH, SOC, Soil Organic Carbon; C/N, Total Organic Carbon: Total Nitrogen; Available N, and N/P, Available N: Available P. Bold values indicate significant differences.

Spearman correlations results revealed that the Chao1 and Shannon index of *A. inebrians* root bacterial communities had no significant (*P* > 0.05) correlation with soil properties (Table 3). Additionally, according to the RDA between the root bacterial community and soil properties, the first and second axis of the plotted RDA results explained 19.3% and 10.3% of the variance, respectively, (Figure 4A). Furthermore, Actinobacteria was positively correlated with soil AN, AP, pH, and TP, while negatively correlated with soil NN, AK, and available N (Figure 4A). Meanwhile, Proteobacteria was positively correlated with soil AK, NN, and available N while negatively correlated with soil TP, pH, AP, and AN (Figure 4A).

**FIGURE 5:**
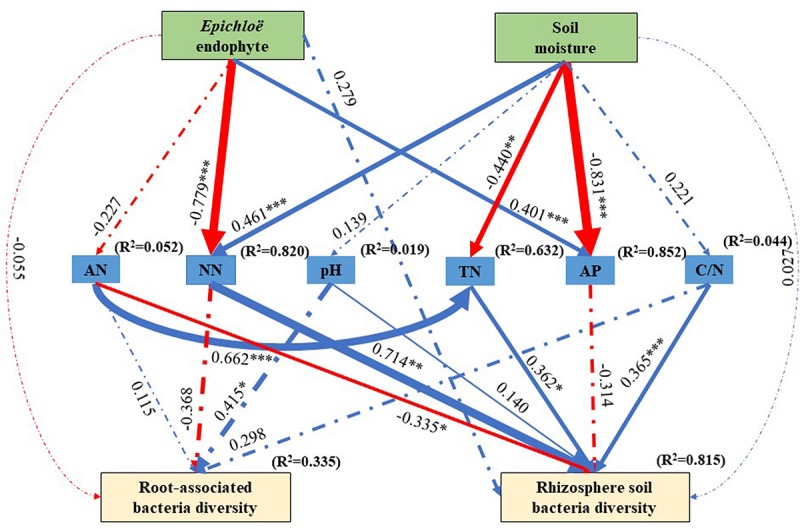
The structural equation modeling showing the causal relationships among *E. gansuensis* endophyte, soil moisture, soil NN (Nitrate Nitrogen), TN (Total Nitrogen), pH, AP (Available P), Available N, C/N (Total Organic Carbon: Total Nitrogen), root-associated, and rhizosphere soil bacterial community diversity. Arrows indicate significant relationships, dotted lines indicate no significant relationships, and solid lines indicate significant effect. The blue line represents the positive effect, red line represents the negative effect. The width of arrows indicates the strength of the causal effect. *R*^2^ values represent the proportion of variance explained for each variable. Model fit summary (χ^2^ = 18.674, Df = 23, *P* = 0.720, NFI = 0.858, and RMSEA = 0.336) are showed. Numbers above arrows indicates path coefficients. ^∗^*P* < 0.05, ^∗∗^*P* < 0.01, and ^∗∗∗^*P* < 0.001.

The best SEM (χ^2^ = 18.674, df = 23, *P* = 0.720, NFI = 0.858, and RMSEA = 0.336) explained 33.5% of the variations in the root-associated bacterial community diversity and 81.5% of variations in the rhizosphere soil bacterial community diversity (Figure 5). *E. gansuensis* decreased the diversity of the root-associated bacterial community and increased the diversity of the rhizosphere soil bacterial community through decreasing soil available N content (Figure 5). Soil moisture increased the diversity of the rhizosphere soil bacterial community through significantly increasing soil NN, and non- significantly increasing pH and C/N, and significantly decreasing soil AP (Figure 5).

## Discussion

Our study that examined the influence of the presence of a mutualistic seed-borne, systemic fungal endophyte and also the effects of different available soil moisture on bacterial communities revealed that *E. gansuensis* influenced the diversity and richness of the bacterial community in the roots and rhizosphere soil of *A. inebrians* plants, and soil moisture only affected the diversity and richness of the bacterial community of *A. inebrians* plants rhizosphere soil. As with some other studies, different soil moistures can have negative, neutral or positive effects on the diversity of both the rhizosphere and the root-associated bacteria (Zhang et al., 2014b; Xu et al., 2018). In general, the diversity of both the rhizosphere and the root-associated bacteria tended to be lowest under low moisture (Xu et al., 2018). Our study also indicated that the presence of *E. gansuensis* in *A. inebrians* plants decreased the diversity of the root-associated bacterial community, but enhanced the diversity and richness of the rhizosphere soil bacterial community.

### Diversity of Root and Rhizosphere Soil Bacterial Communities

The previous studies on the diversity in the bacterial community in roots and the rhizosphere soil, using different plants and different growing conditions, found that as for our findings, the rhizosphere soil had a higher diversity than actually in the roots. Donn et al. (2015) found that the composition of bacterial communities in roots of wheat plants and rhizosphere soils showed obvious differences, and the diversity of these bacterial communities decreased from soil to roots. Edwards et al. (2015) also found a similar pattern in rice, which supported the conclusion that bacterial microbial diversity in soil was higher than that in roots. In contrast to these findings was the one that looked at the rhizosphere and root-associated bacteria in *Am. breviligulata*, a grass that thrives in sand dunes. The bacterial diversity and richness in the root system of this grass was significantly higher than in the soil, and it may be that root exudates offer much-needed resources for root bacteria than are present in the sandy soil in dune ecosystems (Bell-Dereske et al., 2017). Bulgarelli et al. (2012) revealed that Actinobacteria and Bacteroidetes are the dominant phyla in *Arabidopsis* roots and rhizosphere soils. And then, Schlaeppi et al. (2014) found that the dominant bacterial populations in roots and soil microbiota of *Ar. thaliana* are Actinobacteria, Bacteroidetes and Proteobacteria. Our current experimental results showed that Actinobacteria and Proteobacteria were the most abundant phyla of bacterial communities in roots and rhizosphere soil, which indicated that genera of Actinobacteria and Proteobacteria may be key bacteria in the root and rhizosphere.

### Effects of *Epichloë* Endophyte on the Belowground Bacteria Community

Our continuing studies are being conducted to investigate the effects of aboveground *Epichloë* endophytes on belowground microbial communities associated with *A. inebrians* host plants. As part of these studies Zhong et al. (2018) showed that the presence of an *Epichloë* endophyte reduced the diversity of root-associated fungal communities associated with *A. inebrians*. Studies on other plant species/*Epichloë* endophytes associations have also investigated these effects and these are valuable comparative studies to compare and contrast with our studies. The study by Bell-Dereske et al. (2017) indicated that the presence of an *Epichloë* endophyte in *Am. breviligulata* decreased the diversity of the root-associated bacteria community under elevated soil moisture. Similarly, our present study indicated that *E. gansuensis* decreased the Shannon diversity of the root-associated bacterial community of *A. inebrians*. The presence of an *Epichloë* endophyte altered the composition of the soil bacterial community associated with *Lolium multiflorum*, while having no apparent effect on the soil fungal community (Casas et al., 2011). Roberts and Ferraro (2015) showed that the *Epichloë* endophyte of tall fescue increased the rhizosphere soil bacteria diversity. Our study similarly found that *E. gansuensis* enhanced the diversity and richness of rhizosphere soil bacteria community.

Some previous studies had shown that the effects of *Epichloë* endophytes on belowground bacteria may be caused by secondary metabolites (Vandegrift et al., 2015; Rojas et al., 2016; Soto-Barajas et al., 2016), such as root exudates (Guo et al., 2015), alkaloids (Franzluebbers and Hill, 2005), and root volatile organic compounds (Rostás et al., 2015). A study had demonstrated that an *Epichloë* endophyte altered the composition of root exudates, such as the total phenolic content and TOC (Guo et al., 2015); in addition, studies also found that root exudates could construct the belowground bacterial community (Badri and Vivanco, 2009; Bakker et al., 2013). Although studies have been conducted on the content of alkaloids in the aboveground tissue of *A. inebrians* (Zhang et al., 2011, 2014a), the presence of these alkaloids in the roots and rhizosphere soil of *A. inebrians* has not been reported. Previous studies also showed that *Epichloë* endophytes produced changes in soil properties, including soil total nitrogen content (Buyer et al., 2011), inorganic nitrogen (Franzluebbers and Hill, 2005), TOC content (Guo et al., 2015), biomass C (Buyer et al., 2011), and pH (Shen et al., 2013). Our present study also found that *E. gansuensis* decreased soil available N content and this was associated with enhanced diversity of the *A. inebrians* rhizosphere soil bacterial community and decreased diversity of the root-associated bacterial community. Compared with previous findings, our second hypothesis that *E. gansuensis* affect bacterial diversity of *A. inebrians* by changing soil physical and chemical properties was supported by the present study.

### Effects of Soil Moisture on Diversity of the Belowground Bacterial Communities

Previous studies on the effects of precipitation and drought stress on microbial community changes have also revealed that underground microorganisms are affected by soil moisture. According to Xu et al. (2018) proposed that drought stress reduced the diversity of bacterial communities in sorghum-related rhizosphere soil. Naylor et al. (2017) and Santos-Medellín et al. (2017) also highlighted that drought stress changed the composition of bacterial communities associated with rice and some grass/crop species. In our present study, compared to the normal soil moisture, drought markedly decreased the richness of the rhizosphere soil bacterial communities of *A. inebrians*, and the well-watered treatment significantly increased the richness of the rhizosphere soil bacterial community, and this supports our first hypothesis that soil moisture could influence the bacterial diversity in roots and rhizosphere soil of *A. inebrians*.

Soil moisture, as a significant contributor to belowground bacterial community changes, has been reported to have profound effects on soil microbial activity, thus affecting carbon input as well as decomposition of soil organic matter, and this will contribute to plant growth (Zhang et al., 2014b; Naylor et al., 2017; Olatunji et al., 2018). Furthermore, previous findings indicated that soil properties are a major driver of differences in the distribution and composition of bacterial communities (Zhang et al., 2014b; Olatunji et al., 2018). These soil properties include physical structure, microbial activity, organic compounds, nutrient transformation and the presence of root exudates (Zhang et al., 2014b; Francioli et al., 2016; Zhalnina et al., 2018). Our current study also found that soil moisture changed the diversity of rhizosphere soil bacteria of *A. inebrians* by increasing soil pH, C/N, and NN content and decreasing soil AP content. This is consistent with previous research results (Shen et al., 2013; Zhang et al., 2014b; Van der Bom et al., 2018). Meanwhile, our results also supported the second hypothesis that different soil moisture treatments led to the changes of soil properties, which in turn can bring changes to the diversity and richness of the soil bacterial community of *A. inebrians*.

### Relationships Between Bacteria Community and Environmental Factors

Previous studies have shown that the diversity and composition of bacterial communities associated with plant roots and rhizosphere soil are affected by a series of biotic and abiotic factors, such as fertility, pH and soil moisture (Shen et al., 2013; Zhang et al., 2014b; Yao et al., 2018). These factors normally lead to changes in the physical and chemical properties of rhizosphere soil and soil nutrient levels, which are closely related to the diversity of rhizosphere soil and root bacteria (Bulgarelli et al., 2012; Fan et al., 2017; Francioli et al., 2017). Meyer et al. (2013) demonstrated that the availability of inorganic nitrogen regulates the relative diversity of bacteria and archaea of soil microbial communities among different types of land use intensity in grassland ecosystem, and bacteria are involved in the whole process of inorganic nitrogen cycling. Van der Bom et al. (2018) also highlighted that the effects of *N* inputs on the soil bacterial community structure in the field was greater than that of *P* or *K* inputs. Nie et al. (2018) revealed and high levels of *N* addition decreased soil bacterial diversity and altered the composition of the forest soil bacterial community. The present study indicated that with decreasing soil available N, the diversity of the root-associated bacterial community was decreased and the diversity of the rhizosphere soil bacterial community was increased. Our study also found that soil *N*, AP, and pH content was closely correlated with rhizosphere soil bacterial diversity of *A. inebrians.* Rousk et al. (2010) also found that the richness and diversity of bacterial communities were positively correlated with pH, and the diversity of the bacterial community almost doubled when pH was increased from 4 to 8. Dimitriu and Grayston (2010) noted that the relative abundance of Acidobacteria increased with lower pH. Meanwhile, Shen et al. (2013) also found that bacterial communities differed sharply at different altitudes, and bacterial community composition is closely related to soil pH, which also emphasized that pH was a good predictor of the diversity distribution of soil bacterial communities at different altitudes. Our present study also demonstrated that soil pH was closely correlated with the diversity and composition of the *A. inebrians* bacterial community, which fully supported our second hypothesis that the presence of *E. gansuensis* and soil moisture treatments can bring changes in soil physical and chemical properties, and a close relationship was observed between underground bacteria and soil properties in our research.

## Conclusion

This study revealed that rhizosphere soil of *A. inebriens* plants harbored a richer and more diverse bacterial community than the roots. In addition, the presence of *E. gansuensis* in *A. inebriens* plants significantly decreased the Shannon diversity of the root-associated bacterial community, and increased the Shannon diversity of the rhizosphere soil bacterial community. In addition, soil moisture increased the Shannon diversity of the rhizosphere soil bacterial community. Meanwhile, Chao1 richness of the rhizosphere soil bacterial community of *A. inebrians* significantly increased with the increase in the soil moisture level. Moreover, the present study also indicated that the diversity and richness of *A. inebrians* root-associated and rhizosphere soil bacterial communities were intimately associated with soil properties of available N, C/N, NN, AP, and pH. Therefore, two hypotheses were proved by our present study. Further experiments should systematically study the mechanism of soil moisture and the aboveground *E. gansuensis* endophyte on the richness and diversity of root and rhizosphere bacterial communities.

## Data Availability Statement

The datasets generated for this study can be found in the Sequence Read Archive (SRA) https://www.ncbi.nlm.nih.gov/sra/?term=PRJNA590316.

## Author Contributions

YJ and XZ designed this experiment. YJ and RZ performed the soil moisture treatment, managed the experimental field. YJ and RZ measured the soil data. YJ and RZ analyzed the data. YJ and XZ wrote this manuscript. MC revised this manuscript and polished the English. All authors contributed to revise the manuscript.

## Conflict of Interest

The authors declare that the research was conducted in the absence of any commercial or financial relationships that could be construed as a potential conflict of interest.

## Footnotes

1 https://www.ncbi.nlm.nih.gov/sra/SRP117302

2 http://qiime.org/scripts/assign_taxonomy.html

3 Release 128 http://www.arb-silva.de
