# Supplementary material for: Effects of Epichloë gansuensis Endophyte on the Root and Rhizosphere Soil Bacteria of Achnatherum inebrians Under Different Moisture Conditions
Source: Front Microbiol. 2020 Apr 17;11:747. doi: 10.3389/fmicb.2020.00747 (PMC7181407; doi:10.3389/fmicb.2020.00747)
Supplement: Supplementary file 1 [file Data_Sheet_1.docx]

Table S1 Root-associated and rhizosphere soil bacterial community composition of *Achnatherum inebrians* under different soil moisture and endophyte treatments at the [phylum](https://fanyi.so.com/#phylum) level (n=3, D: drought, N: normal, W: well-watered, EI: endophyte-infected and EF: endophyte-free).

| Samples | Phylum | DEI | DEF | NEI | NEF | WEI | WEF | OTUs | Sequences |
| --- | --- | --- | --- | --- | --- | --- | --- | --- | --- |
| Root | Acidobacteria | 0.11 | 0.21 | 0.35 | 0.29 | 0.28 | 0.20 | 356 | 85530 |
|  | Actinobacteria | 29.06 | 36.08 | 35.40 | 26.10 | 30.27 | 24.67 | 583 | 628812 |
|  | Bacteroidetes | 2.02 | 1.01 | 2.04 | 2.41 | 3.73 | 3.84 | 235 | 108099 |
|  | Chloroflexi | 0.28 | 0.46 | 0.73 | 0.82 | 1.03 | 0.65 | 421 | 69190 |
|  | Cyanobacteria | 23.81 | 20.18 | 20.70 | 30.21 | 21.25 | 20.67 | 67 | 470427 |
|  | Firmicutes | 19.02 | 17.18 | 13.93 | 14.21 | 15.14 | 16.40 | 329 | 360241 |
|  | Fusobacteria | 0.07 | 0.53 | 0.74 | 0.13 | 0.22 | 2.01 | 103 | 101388 |
|  | Gemmatimona | 0.03 | 0.08 | 0.07 | 0.05 | 0.05 | 0.04 | 77 | 15035 |
|  | Proteobacteria | 23.41 | 23.05 | 23.77 | 23.09 | 23.42 | 27.35 | 124 | 494883 |
|  | Saccharibacteria | 0.09 | 0.13 | 0.09 | 0.06 | 0.62 | 0.15 | 75 | 13689 |
|  | others | 2.10 | 1.09 | 2.18 | 2.63 | 3.99 | 4.02 | 257 | 137329 |
|  | Total | 100 | 100 | 100 | 100 | 100 | 100 | 2627 | 2494623 |
| Soil | Acidobacteria | 6.35 | 7.23 | 8.18 | 5.91 | 7.64 | 9.13 | 672 | 90212 |
|  | Actinobacteria | 26.05 | 25.28 | 20.59 | 23.19 | 15.91 | 16.92 | 814 | 406801 |
|  | Bacteroidetes | 3.09 | 2.23 | 2.70 | 2.33 | 2.81 | 2.81 | 283 | 24410 |
|  | Chloroflexi | 10.16 | 10.14 | 12.74 | 9.14 | 10.68 | 10.89 | 314 | 113983 |
|  | Cyanobacteria | 4.20 | 4.05 | 2.93 | 8.51 | 4.38 | 0.21 | 662 | 93005 |
|  | Firmicutes | 18.35 | 15.67 | 15.71 | 14.39 | 16.46 | 21.92 | 483 | 294269 |
|  | Fusobacteria | 0.73 | 3.22 | 2.83 | 5.56 | 3.82 | 7.01 | 192 | 96022 |
|  | Gemmatimona | 0.31 | 3.50 | 4.00 | 3.50 | 4.71 | 0.24 | 325 | 78446 |
|  | Planctomycetes | 0.99 | 1.06 | 1.02 | 0.88 | 1.27 | 1.16 | 124 | 15928 |
|  | Proteobacteria | 21.98 | 21.42 | 21.50 | 21.09 | 23.04 | 20.81 | 268 | 516904 |
|  | Saccharibacteria | 1.06 | 0.82 | 1.17 | 0.95 | 1.06 | 0.90 | 219 | 31534 |
|  | Others | 6.73 | 5.29 | 6.63 | 4.55 | 8.22 | 8.00 | 638 | 115242 |
|  | Total | 100 | 100 | 100 | 100 | 100 | 100 | 4994 | 1876756 |


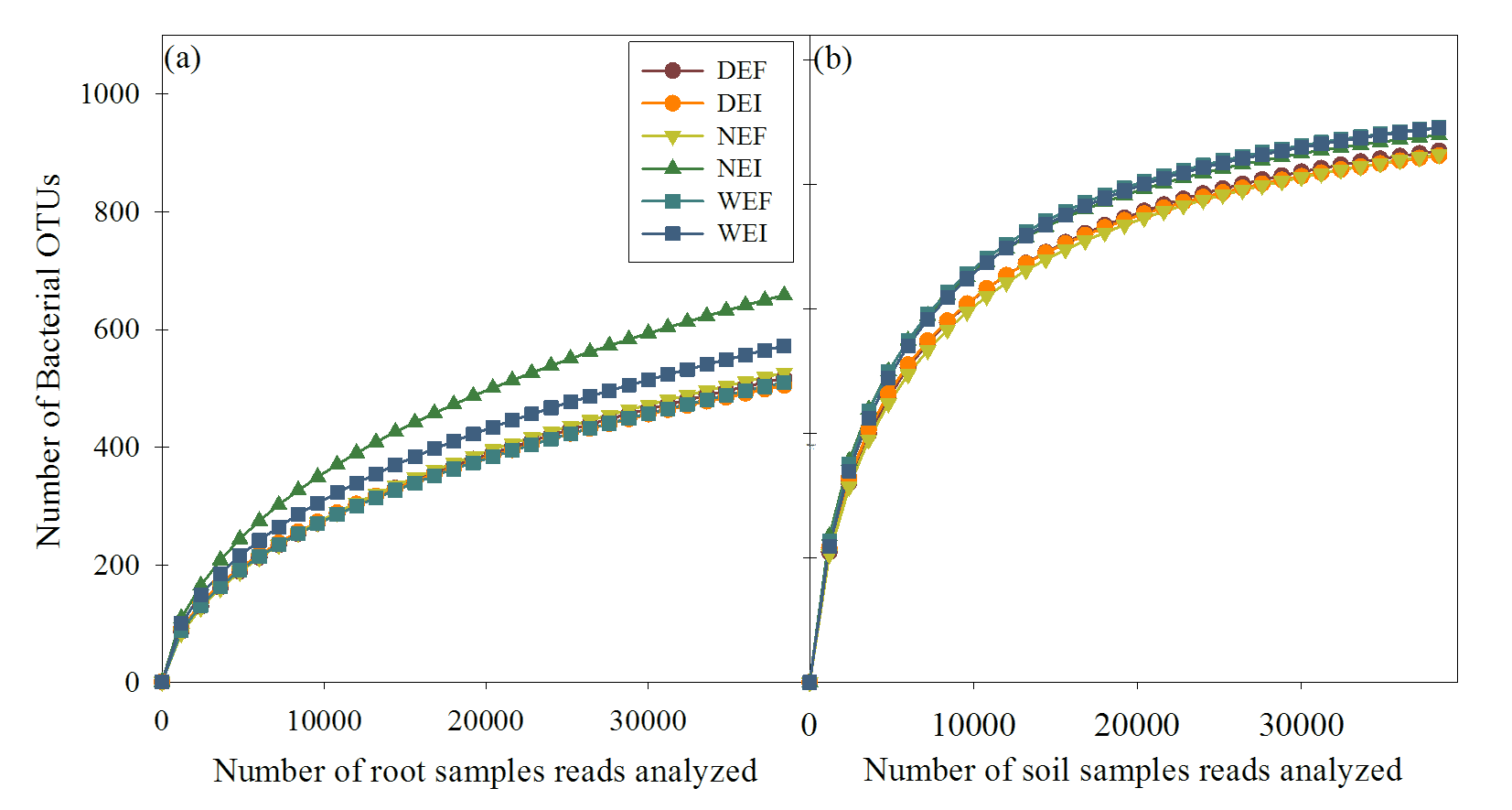


Figure S1

Rarefaction curves of the bacterial 16S rRNA gene sequence sampled from the roots (a) and rhizosphere soil (b) of *Achnatherum inebrians* under different soil moisture and endophyte treatments (n=3, D: drought, N: normal, W: well-watered, EI: endophyte-infected and EF: endophyte-free).
